# Autologous Hematopoietic Stem Cell Transplantation for Treatment of Systemic Sclerosis

**DOI:** 10.3389/fimmu.2018.02390

**Published:** 2018-10-16

**Authors:** Nicoletta Del Papa, Francesca Pignataro, Eleonora Zaccara, Wanda Maglione, Antonina Minniti

**Affiliations:** Dipartimento di Fisiatria e Reumatologia, Istituto Ortopedico Gaetano Pini, Milan, Italy

**Keywords:** systemic sclerosis (Scleroderma), stem cells, therapy, transplantation, haematopoietic

## Abstract

Systemic Sclerosis (SSc) is a complex autoimmune disease, characterized by high mortality and morbidity. The heterogeneity in terms of extent, severity, and rate of progression of skin and internal organ involvement gives rise to many difficulties in finding the optimal therapeutic interventions for SSc and, to date, no disease-modifying agents are available. In this scenario, it is not surprising that SSc was one of the first autoimmune diseases challenged with high-dose immunosuppressive treatment followed by autologous hematopoietic stem cell transplantation (AHSCT). In the last decades, AHSCT has emerged as a treatment option for refractory SSc through a reduction of the aberrant immune cells, followed by re-constitution of a new, self-tolerant immune system. After several case series and pilot studies, more recently three randomized controlled trials have shown a benefit in skin involvement, organ functions and quality of life measures in AHSCT compared to monthly cyclophosphamide. In addition, although AHSCT presents a certain risk of mortality, it has been shown that the overall survival is better, compared to the cyclophosphamide group. Current evidence suggests that SSc patients who are most likely to benefit from AHSCT are early, active, with rapidly progressing diffuse skin disease, and mild involvement of internal organs. As the studies have progressed, it has become evident the need for a more rigorous patient selection, the optimization of transplant and post-transplant procedures, and the intervention of multidisciplinary teams of specialists to increase the safety and efficacy of AHSCT in SSc.

## Introduction

Hematopoietic stem cell transplantation, commonly used to treat hematological malignancies, has evolved in the last 20 years as a specific therapy for severe and therapy-refractory autoimmune diseases. Since the first case series, and following prospective and retrospective studies [recently reviewed by Eyraud et al. (1)] reporting the feasibility and efficacy of AHSCT in SSc on skin thickening and stabilization of internal organ function, randomized controlled trials have recently provided evidence that AHSCT is a real disease-modifying treatment for diffuse SSc inducing better long-term survival in comparison with intravenous cyclophosphamide (2–4). These data have provided convincing proof of the superiority of AHSCT over conventional therapies even considering the intrinsic risk of the procedure. Really, the risk of transplant-related mortality (TRM) has decreased over the years (5) thanks to the careful selection of patients, growing experience in organ pre-transplant screening and ongoing refining of transplant procedures. Although AHSCT has been proved to be effective in patients with SSc, more in-depth knowledge about the mechanisms of action related to AHSCT-induced remission is required. AHSCT is considered a sort of intense immune-suppressive therapy able to ablate the aberrant auto-reactive immune cells to allow for a subsequent resetting of the host immune system. Indeed, the effects of AHSCT are now believed to be more complex, due in part to the intense immune ablative conditioning regimen, the modulation of mediators of innate immunity and adaptive immune cells, and the regenerative effects of the infusion of hematopoietic progenitors on damaged tissues (6).

In this review, we will examine the outcome of conventional therapy of SSc, provide a detailed description of the procedure, the clinical results and risks of AHSCT for SSc. We will present a comparison of the design and outcomes of published randomized trials regarding AHSCT in SSc. We will further discuss the options and recommendations for a better pre-transplant selection and evaluation of SSc patients to refer for AHSCT. We will also provide an analysis of the actual unmet needs in AHSCT and the issues that determine why AHSCT is still seen as a rescue therapy rather than an effective therapy for long-term suppression of SSc.

## Current immunosuppressive management of SSc

SSc is an immune-mediated disorder characterized by inflammatory, vascular, and fibrotic features resulting in skin fibrosis and multiple organ manifestations (7). Despite the fact that in the last 20 years there has been improved understanding in the early diagnosis of the disease, and in identifying early internal organ involvement, therapy is still an unsolved problem (8, 9). While some patients display an indolent course, others exhibit a rapid and severe progression of the fibrotic processes with early manifestations of vital organ dysfunction. The lack of validated biomarkers that could be used for diagnosis, disease classification, identification of probable organ involvement and evaluation of therapeutic response, increases the difficulties in the management of scleroderma patients (10). The ability to identify different clinical phenotypes with a heterogenous course, to recognize the existence, like other autoimmune diseases, of flare and remission phases, and to estimate the prognosis of the disease would also be of remarkable importance from different points of view. In this perspective, the therapeutic strategies to treat SSc patients should not be addressed to a simple “organ-based” treatment, but to a more complex evaluation of the patient aimed at the prompt detection of active phases of the disease, assessment of possible organ damage and patient prognosis.

Current treatment options for SSc have targeted different pathogenic processes, including inflammation, immune dysregulation and fibrosis, showing only limited efficacy, and, to date, SSc still continues to carry a very high morbidity and mortality rate, mainly in the rapidly progressive form of the disease (11, 12). For a long time, cyclophosphamide has been considered a first line therapy for SSc, namely in patients with skin disease and concomitant interstitial lung disease (ILD) (13). More recently, two randomized, placebo-controlled trials involving patients with SSc showed that mycophenolate and cyclophosphamide were effective against ILD associated with SSc and, in particular, mycophenolate was useful in terms of tolerability, improvement of lung function and dyspnea, thickening of the skin, and health-related quality of life. However, all the effects, except for a sustained impact on dyspnea, disappeared approximately 1 year after stopping oral administration of cyclophosphamide (14–19). Furthermore, two meta-analyses of prospective studies using oral or IV cyclophosphamide in SSc-related ILD did not report any improvement in lung function (20, 21).

With regard to other immunosuppressive agents, a wide variety of treatments for SSc have been explored including azathioprine, methotrexate and, more recently, targeted therapies with monoclonal antibodies, including tocilizumab (anti-interleukin-6 receptor antibody), rituximab (an anti-CD20 antibody), and fresolimumab (an anti-TGFß antibody) (22). However, although showing a possible improvement of skin and lung involvement, they have been used in small series, pilot or short-term studies without yielding any definite evidence that they may be effective in changing the natural history of scleroderma disease (9, 19). Based on this uncertainty and frustration, AHSCT has been seen as a hopeful opportunity to treat SSc.

### AHSCT procedure

The AHSCT procedure is based on three main steps: the first one consists of the mobilization of stem cells from bone marrow to the peripheral blood by priming regimens followed by the collection of the mobilized stem cells; the second step is the administration of conditioning regimens with an immunoablative or myeloablative effect and, finally, the infusion of autologous (CD34+) stem cells (so called “transplantation”).

The mobilization protocols include the administration of cyclophosphamide (2–4 gr/m^2^) in combination with granulocyte-colony stimulating factor (G-CSF). Mobilized stem cells are then harvested by leukapheresis with or without CD34 selection before cryopreservation. Before transplantation, the patient undergoes the conditioning phase that can be either non-myeloablative or myeloablative. The goal of non-myeloablative regimens is to maximally suppress the immune system without destroying the bone marrow stem cell compartment. The bone marrow is not completely wiped out, which makes the treatment less dangerous and allows the patient to recover faster. Myeloablative AHSCT is the more stringent type of treatment. The term myeloablation refers to the administration of total body irradiation (TBI) and/or alkylating agents, at doses which will not allow autologous hematologic recovery. This is designed to almost completely wipe out both the autoreactive lymphocytes, as well as the bone marrow. Both forms are equal in that they both ablate the lymphocytes in the body that are self-intolerant, and are ultimately responsible for the underlying autoimmune disease. However, current evidence supports the concept that non-myeloablative AHSCT has an improved level of safety and tolerability with lower mortality when compared to myeloablative regimens (23). In this regard, it is well known that in hematological diseases TBI is associated with the occurrence of secondary myelodysplasia and acute myeloid leukemia and solid tumors, even many years after the treatment (24, 25).

The available data do not allow making conclusions as to which autologous transplant regimen is best since head-to head studies have not been performed. To date, a few studies analyze the features and outcomes of mobilization in patients with autoimmune diseases and particularly with SSc. The use of G-CSF alone has been frequently associated with flare of disease activity in different autoimmune diseases, so the combination with cyclophosphamide is considered advantageous not only in protecting against disease flares but reducing the number of autoreactive T cells in the graft (26–30). With regard to cyclophosphamide dosage, doses used for mobilization in patients with malignancies could not be the best regimen for patients with autoimmune diseases. In patients with SSc and visceral involvement, mobilization-related complications, particularly cardiotoxicity, hemorrhage as well as infections, are largely dependent from cyclophosphamide doses (31–33). Besides the mobilization regimen, stem cell yields could vary according to the underlying disease and prior medication history. There are limited reports investigating the efficacy of stem cell mobilization and harvesting in patients with autoimmune diseases treated by AHSCT. In general, patients with SSc have not shown any relevant difference in terms of progenitor mobilization in comparison with patients suffering from other autoimmune disease and no correlation has been observed with respect to disease duration and previous exposure to methotrexate, cyclophosphamide or prednisone (26, 34, 35). With regard to the selection of cells for the graft, a recent multicenter retrospective study has demonstrated that the use of selected CD34+ cells for AHSCT in patients with SSc did not add any benefit to the outcome with respect to the use of un-manipulated cells (36).

## Immunologic mechanisms of AHSCT

SSc is considered a pleiomorphic disease deriving from the complex interaction of endothelial damage, autoimmune inflammatory reaction and excessive fibrosis, and is related to Th1/Th2 dysregulation, with prevalence of Th2 cells and development of autoreactive T and B cells targeting self-antigens causing organ damage (7). Considering this scenario, hematopoietic stem cell transplantation aims to reconstitute the hematopoietic niche after chemotherapy treatment or irradiation to obliterate autoreactive cells.

The result of this procedure is an “immune resetting,” that is the eradication of the preexisting immune system, which is replaced by a new immune repertoire, with re-instatement of an appropriate immune regulation. These three well-defined mechanisms of immune resetting may be synergistic and their relative contribution to disease control depends on the transplantation regimen and on the underlying disease (37).

The combination of lymphotoxic chemotherapy, such as cyclophosphamide and anti-thymocyte globulin, leads to a profound and long-lasting lymphopenia and persistently reduced levels of putative pathogenic autoantibodies. Apart from this non-specific immunosuppression, there is growing evidence that autologous AHSCT can also re-establish immunological tolerance through different mechanisms. Firstly, AHSCT leads to an increased number of regulatory, FoxP3+ T cells, which are important in the preservation of tolerance (38). Secondly, the reactivation of thymic function after autologous AHSCT potentially leads to a tolerant, “juvenile” immune system. This has been illustrated by the recurrence of recent thymic emigrating cells, characterized by T-cell receptor excision circles (TREC) and CD31 expression, reestablishing T-cell receptor diversity in the years after AHSCT in patients with SLE (39). Moreover, anti-thymocyte globulin directly targets long-living, autoantibody-producing plasma cells by complement mediated lysis and apoptosis (40).

The immune reconstitution post-therapy results in profound changes of circulating immune cell populations, which involve a functional reactivation and volumetric enlargement of the thymus, defined as thymic rebound. However, the presence of pre-transplant B cell clonal expansion and faster T cells recovery after transplantation represent specific immunologic characteristics of long-term non-responder/relapsing patients.

### T cells and T cell receptor repertoire

The cytopenia following the conditioning phase of AHSCT affects the lymphocyte subsets differently, and the kinetics of reconstitution depends on different timing of recovery for each cell type. The levels of B cells, natural killer cells (NKs), and CD8+ T cells begin raising rapidly and achieve a complete reconstitution to pre-transplantation levels after 2–3 years. The recovery of CD4+ T cells has consistently been observed to be slower and is often incomplete. Particularly, delayed CD4+ T cells recovery appears more pronounced in SSc patients who present good response after HSCT in comparison to poor responders (41). When compared with pre-transplant levels, absolute regulatory T cells (Treg) numbers increased significantly at 12 months post-transplantation, concurrent with thymic rebound (42). It is noteworthy that good responders to transplant present higher CD4+CD25^high^FoxP3+ Treg percentages than non-responders (42). This data suggests that these regulatory molecules probably play an important role in the renewal of the immune system. Furthermore, increase of PD-1+ (programmed death-1 positive) T-cell numbers has been described in a group of SSc patients with better outcomes after AHSCT (42). PD-1 expression is critical to induce self-tolerance in newly generated T-cells under lymphopenic conditions, and its absence is associated with development of a systemic multi-organ inflammatory disease (43). In other words, PD-1 positivity in responders suggest an additional mechanism of negative regulatory control on autoreactive pathogenic T-cells (44, 45).

The influx of newly generated thymic-derived naïve T-cells results in changes in the T-cell receptor (TCR) repertoire following transplantation. In the early period post graft, TCR repertoire has been demonstrated to be disturbed with a higher number of families presenting a skewed and oligoclonally expanded profile. Later, thymic rebound results in elevated TCR repertoire. Estimation of TCR diversity showed that, for responder patients, overall specificities increased following thymic rebound at 1 and 2 years, whereas non-responder patients failed to achieve higher TCR diversity (46).

Although AHSCT includes high dose immunosuppression, potentially pathogenic T cells are not completely depleted, because specific T-cell clones can still be detected post graft (47). There are two sources to consider for persistent autoreactive clones after autologous HSCT: residual clones in the host resisting after the immunoablative therapy, and cells reinfused with the graft. The intensity of the immunosuppressive regimen (myeloablative, high-dose or non-myeloablative, reduced intensity conditioning) and the manipulation of the graft (enriched in stem cells or unmanipulated), are the main factors that determine the number of residual T cells (37).

The new immunological arrangement after grafting will depend on the different antigenic stimuli, on the affinity for the ligand and the function of the responding cells (effector or regulatory), which are reprogrammed during the immune system reset.

### B cells

Naïve B-cell counts progressively begin increasing from the sixth month after AHSCT. Particularly, SSc patients with good response to transplantation presented a sustained B cell reconstitution compared to non-responders. Percentage and absolute numbers of CD24^high^CD38^high^ Bregs increase significantly in the following months post-AHSCT and responders are shown to present significantly higher frequencies of Bregs than non-responders and Breg levels correlate with a favorable outcome in SSc patients (42).

It should be mentioned that thymic rebound, as well as increased bone marrow output of newly generated naïve B cells, are exclusive of the post-transplant setting and are not observed in SSc patients receiving conventional treatment (42).

### Autoantibodies

To date, literature data about the presence and the modification of anti-Scl70 antibodies after AHSCT are inconclusive. Some studies suggest a correlation between anti-Scl70 titer and clinical response, whereas others show uncertain association (48–50). Henes and colleagues demonstrated the anti-Scl70 reactivity significantly decreased after transplantation but remained positive in 10 of the 11 patients followed for up to 24 months. This decrease did not correlate with the clinical outcome after grafting (49). Farge et al. reported long-term anti-Scl70 negativity after transplantation in responder patients, although this decrease was not associated with a reduced B cells counts (46). Recently Glaeser et al. recognized a specific epitope recognized by anti-topo-1-antibodies in SSc sera. Interestingly, SSc patients with a good response to AHSCT had lower reactivity towards this peptide (p39, aa647-671) in comparison to non-responders (50).

### Cytokines

The immune reconstitution process following the AHSCT may involve also the serum levels of the inflammatory cytokines profile and pro-fibrotic molecules concentration.

Detection of circulating and tissue cytokine levels has provided evidence for a balance between Th1/Th2 cytokines in the course of SSc, supporting a predominant Th2 immune response (7, 51, 52). A correlation between cytokine levels and SSc severity, in terms of extent of skin and organ fibrosis, has been widely reported (52), suggesting cytokines as a target of new therapeutic strategies, including AHSCT.

Currently, three studies examined the evolution of serum cytokine profile in SSc patients underwent AHSCT. The first study analyzed the serum levels of inflammatory cytokines (IL-2, IL-6, IL-8, and IFN-γ), pro-fibrotic molecules (TGF-β, IL-4, and PDGF), pro-angiogenic factors (VEGF and PDGF), endothelial markers (E-selectin and P-selectin) and MCP-1 chemokine before and up to 4 years after HSCT in 20 SSc patients. Even though a decrease in IL-2 and IL-8 levels, along with a slight but significant decrease in TGF-ß levels after 6 months has been demonstrated, these fluctuations did not reflect the skin score improvement after AHSCT (53).

In a cohort study of 11 patients, the concentrations of tumor necrosis factor alpha (TNF-α), soluble Interleukin 2 receptor (sIL-2r) and IL-6 levels were detected 12 months after AHSCT. Levels of TNF-α, sIL-2r, and IL-6 were significantly decreased, although reached normal values after 3 and 6 months post-AHSCT. TGF-β1 titers were not statistically significant decreased. Serum levels of vascular endothelial growth factor (VEGF) and monocyte chemoattractant protein-1 (MCP-1) did not decrease (48).

Assassi et al. analyzed gene expression patterns in the peripheral blood from patients participating in the SCOT Trial (4), and showed that the interferon signature was decreased by AHSCT. SSc patients presented a significant up regulation of genes that are induced by IFN. After 26-months of follow up, the IFN transcript score decreased significantly in patients receiving grafting, whereas it remained stable in the patients treated with monthly cyclophosphamide. Although this may support the “resetting” hypothesis, it remains to be investigated whether the IFN signature remains durably suppressed in the long term (54).

Recent studies have tried to understand if there is an immunological signature characterizing and predicting the clinical response to AHSCT (Table 1), but the findings are not univocal and sometimes confusing. In this subset, further immune reconstitution analysis will guide the clinicians for establishing new targeted therapeutic protocols.

**Table 1:** Immunological profile in patients responders to AHSCT.

**Before and after AHSCT**	**After AHSCT**
- High number of T-reg and B-reg - Broad T-cell receptor diversity	- High number of PD-1+ T cells - Low reactivity to a specific epitope recognized by anti-topo-1-antibodies - Delayed CD4+ T cells recovery

*AHSCT, Autologous haematopoietic stem cell transplant; PD-1, programme death-1*.

## Clinical use of AHSCT in SSc

### Efficacy

Several case reports and different phase I-II studies formed the basis for randomized controlled studies (RCTs) with AHSCT in SSc (1–4). All the RCTs have similar eligibility criteria and control treatment but exact comparability of cohorts, procedures and outcomes is questionable (see Tables 2, 3).

**Table 2:** Overview of study characteristics, inclusion and exclusion criteria of ASSIST, ASTIS and SCOT trials.

	**ASSIST (2)**	**ASTIS (3)**	**SCOT (4)**
Trial design	Randomized Phase II	Randomized Phase III	Randomized Phase III
Patients number	19	156	75
Recruitment period	2006–2009	2001–2009	2006–2011
Mobilization	CYC 2 g/m^2^, G-CSF	CYC 4 g/m^2^, G-CSF	G-CSF only
Conditioning	CYC (200 mg/kg), rabbit ATG	CYC (200 mg/kg), rabbit ATG	CYC (120 mg/kg), equine ATG
Total body irradiation	No	No	Yes (800 cGy, lung and kidney shielding)
Stem cell manipulation	None	CD34+ selection	CD34+ selection
Comparator arm	CYC 6 monthly IV courses (1,000 mg/m^2^)	CYC 12 monthly IV courses (750 mg/m^2^)	CYC 12 monthly IV courses (750 mg/m^2^)
Inclusion criteria	<60 years	18–65 years	18–69 years
	Diffuse SSc	Diffuse SSc	Diffuse SSc
	Disease duration ≤ 4 years	Disease duration ≤ 4 years	Disease duration ≤ 4 years
	mRSS ≥ 15	mRSS ≥ 15	mRSS ≥ 16
	Internal organ involvement	Internal organ involvement	Internal organ involvement
Exclusion criteria	Mean PAP > 25 mmHg or PAP sys > 40 mmHg	Mean PAP > 50 mmHg	Mean PAP >30 mmHg
	LVEF < 40%	LVEF < 45%	LVEF < 50%
	–	–	FVC < 45% predicted DLCO < 40% predicted
	Creatinine > 177 umol/L	Creatinine Clearance < 40 mL/min	Creatinine Clearance < 40 mL/min
	CYC >6 g IV	CYC cumulative IV dose >5 g or >3 g months oral	CYC cumulative IV dose >3 g/m^2^ or >4 months oral or >6 months IV
	–	–	Active GAVE

*ASSIST, American Scleroderma Stem cell versus Immune Suppression Trial; ASTIS, Autologous Stem cell Transplantation International Scleroderma Trial; SCOT, The Scleroderma Cyclophosphamide Or Transplantation; SSc, systemic sclerosis; CYC, cyclophosphamide; ATG, antithymocyte globulin; mRSS, modified Rodnan skin score; LVEF, left ventricular ejection fraction; PAP, pulmonary arterial pressure; IV, intravenous; GAVE, gastric antral vascular ectasia*.

**Table 3:** Comparison of outcomes among ASSIST, ASTIS, and SCOT.

	**ASSIST (2)**	**ASTIS (3)**	**SCOT (4)**
Primary outcomes measures	>25% decrease in mRSS, or >10%% increase in FVC at 12 months	Survival without new onset of heart, lung or kidney failure	Global Rank Composite Score at month 54
Follow up	2.6 years (mean)	5.8 years (median)	Up to 4.5 years
12-months treatment related mortality in comparator arm	0 (0%)	0 (0%)	0 (0%)
12-months transplant-related mortality	0 (0%)	8 (10.1%)	1 (3%)
Overall mortality	0 (%)	19 (24%)	3 (9%)
P/EFS	80% (2.6 years)	81% (4 years)	79% (4.5 years)

*ASSIST, American Scleroderma Stem cell vs. Immune Suppression Trial; ASTIS, Autologous Stem cell Transplantation International Scleroderma Trial; SCOT, The Scleroderma Cyclophosphamide Or Transplantation; mRSS, modified Rodnan skin score; FVC, forced vital capacity; P/EFS, progression/event free survival without mortality, relapse or progression of the disease*.

The ASSIST is a single-center randomized phase II study, whereas the others (ASTIS and SCOT) are multicenter randomized phase III studies (2, 3, 4). In addition, the ASSIST is a treatment failure study and allowed a cross over to transplant due to unsatisfactory response to cyclophosphamide in eight out of nine control patients (2). The ASTIS and SCOT are survival studies and cross over from the control arm to transplant because of disease progression was not allowed in SCOT, except in the case of 2 years of cyclophosphamide therapy in the ASTIS trial (3, 4). Two trials are non-myeloablastive (ASSIST and ASTIS) using cyclophosphamide at 200 mg/kg in the conditioning phase, whereas the SCOT has used a TBI-based myeloablative regimen with cyclophosphamide 120 mg/kg. Two RCTs used the CD34+ selection of the graft, while the ASSIST did not. The primary end-point of the ASSIST trial was improvement in the modified Rodnan skin score (mRSS) or in pulmonary forced vital capacity (FVC) (2). By contrast, the ASTIS trial had event-free survival (EFS) as the primary end point and the treatment-related mortality (TRM) and toxicity, and progression-free survival as major secondary end points (3). The ASTIS and SCOT trials were initially matched in terms of entry criteria, control arms and end points (3, 4). In 2010, the primary end point of the SCOT trial was changed to a non-clinical outcome, the global rank composite score (GRCS). The GRCS ranked subjects based on a hierarchy of several important outcomes, including mortality, EFS (without organ failure), lung function, scleroderma health assessment questionnaire and the mRSS (4). Composite endpoints are increasingly used as primary efficacy measures in several clinical trials to capture a comprehensive picture of the treatment effect and to improve trial efficiency by increasing the event rate and reducing the sample size required. However, there are also some limitations to use this methodology. First, a true survival curve (e.g., Kaplan-Meier estimate) cannot be obtained for the composite outcome score since all the events that occur throughout the trial are shown. Furthermore, the overall global rank comparison may be statistically significant, though the individual components used for the primary analysis may not be significantly different between the two arms. In addition, confirmation of their validity is needed before they can achieve widespread acceptance and, in the case of SSc trials, they have never been used and validated.

Despite all these differences, the outcome data from these RCTs definitely support the greater benefit of AHSCT in comparison to cyclophosphamide for severe SSc (2, 3, 4). All the patients in the AHSCT arm experienced a significant improvement in the mRSS and functional capacity (HAQ, Disability Index). These results are consistent with previous observations from observational, pilot studies and registries that showed a marked impact of AHSCT on skin thickness (1). At the moment, no other studies on therapeutic intervention in diffuse scleroderma have shown to be so effective in stopping and reversing scleroderma skin involvement. The impressive efficacy of AHSCT on skin thickening has to be regarded as an important issue since high mRSS values have been recognized to be a predictor of poor prognosis and high mortality and, on the contrary, improvement in skin thickness is associated with better survival (55, 56).

Although showing some statistically significant improvement in FVC at 1 year and 2 year, AHSCT has yet to demonstrate a clear clinical significant improvement in DLCO and total lung capacity in all three RCTs, confirming the data from observational and retrospective studies (33, 57–59). With regard to DLCO, in a retrospective case-control study Del Papa et al. reported that the cumulative probability that DLCO values may fall under 50% is lower in the AHSCT group compared to the control group (60). In addition, in a cohort of 89 patients transplanted according to the ASSIST protocol, Burt et al. observed that DLCO was not improved significantly after AHSCT, but when the patients were stratified according to the pre-AHSCT echocardiogram and electrocardiogram characteristics, the DLCO was improved in a subgroup of patients with normal cardiac features, raising the question of the relationship between DLCO and cardiac function (33). Using high resolution computed tomography (HRCT), Launay et al. assessed lung involvement after AHSCT in a small group of SSc patients. The extent of SSc lung fibrotic involvement on HRCT rapidly but transiently regressed 6 months after AHSCT. However, longer-term follow-up showed that the impressive early treatment effects of AHSCT on the extent of pulmonary fibrosis decreased over time and were transient in some patients, returning to the pre-transplant extent two years after AHSCT. Moreover, pulmonary fibrosis appeared to be rather stable up to 60 months of follow-up (61). These data are not surprising since they reflect the known effects of cyclophosphamide on SSc skin and lung fibrosis. In the Scleroderma Lung Study (SLS I), the maximum improvement in lung function was observed at 18 months with 12 months of cyclophosphamide use, but at the end of one additional year off therapy, the beneficial effects on FVC and TLC were lost, whereas the skin score stabilized (16). SLS II showed that immunosuppression with either 2 years of mycophenolate or 1 year of oral cyclophosphamide led to slight improvement in lung function (17, 18). Some additional observational studies showed that a strategy combining IV cyclophosphamide followed by oral maintenance azatioprine or mycophenolate for worsening SSc-ILD was associated with stabilization or improvement of pulmonary function tests in approximatively 50% of patients after 12 months of mycophenolate and 24 months of azatioprine respectively (15, 19). Based on these observations, we could speculate that in the direct comparison between the efficacy of AHSCT and that of cyclophosphamide therapy, the lack of maintenance immunosuppression in the control group might be responsible for better outcomes in the transplanted arm. However, unmet needs exist for post-transplant immunosuppressive treatment and future research should address the question whether an additional post-transplantation management is therefore useful to improve AHSCT outcomes.

Suppression or control of disease activity by AHSCT can be regarded as an additional optimal goal of the AHSCT procedure. A study published in 2017 demonstrated that lowering disease activity can be achieved with AHSCT in a population of SSc patients with high disease activity scores evaluated by a validated scoring system, namely the ESSG by Valentini et al. (60, 62). Furthermore, in the myeloablative trial, only 9% of transplant recipients showed scleroderma relapses by 24 months without any significant changes at 54 months (4). This rate was lower than that observed in the non-myeloablative RCT (3). These results further strengthen the profound effects of AHSCT on the course of the disease characterized by poor prognosis. This is a key issue, since patients presenting a rapidly evolving and active disease could be the best candidates to undergo such an extremely aggressive treatment, and have the best possible results. Indeed, similarly to other rheumatic diseases, the opportunity to early “switch off” the inflammatory and active phases of the disease may offer an opportunity to stop and prevent disease organ damage, finally changing the natural history of an aggressive disease. In this regard, both the long-term RCT studies of AHSCT in SSc showed that EFS and overall survival rates are better for patients in the AHSCT arm than for patients in the control arm (82% at 5 years in the ASTIS and 86% at 54 months in the SCOT) (3, 4). These findings confirmed previous long-term follow-up data from a phase I/II study showing that death from disease progression occurred in 8% of severe SSc patients treated with AHSCT (59). This rate is considerably lower compared to the 5-year mortality rate estimated at 40% in such severe SSc patients (63). Similarly, Del Papa et al. observed a significant reduced disease-related mortality in patients with severe SSc treated with AHSCT in comparison with a historical cohort of age-and sex-matched SSc patients with analogous clinical features treated with conventional immunosuppressive agents (60).

### Safety

Supported by preclinical studies and case reports, and more recently by 3 RCTs providing proof of efficacy of AHSCT over conventional therapies, the uptake of AHSCT in SSc has increased over the last decades and evolved as a specific treatment of patients with the severe rapidly progressive form of the disease (5, 13). This is indeed remarkable when we consider that, among the different severe autoimmune diseases treated with AHSCT, SSc has shown the higher risk of mortality (5). This feature can be explained by the fact that, compared with other autoimmune diseases, SSc patients have an involvement of vital organ function resulting in poor tolerance of AHSCT. However, the risks and adverse effects of AHSCT in SSc have changed over the last 20 years.

#### Adverse effects

Expected complications related to the intense AHSCT-related immunosuppression, are opportunistic infections, urinary infections, neutropaenic fever and viral reactivation (5, 64). They represent the leading cause of mortality after AHSCT for autoimmune disease (64, 65) and cluster within the first month after AHSCT. In the ASTIS trial, viral infections were detected in 27.8% of patients in the AHSTC group *versus* 1.3% in the control arm (3). Conversely, overall infection rates were similar in the two arms of the SCOT trial with the exception of varicella zoster infection that developed in 12 out of 33 transplanted patients (36%) (4).

AHSCT can also induce other off-target adverse effects including transient alopecia and amenorrhea, and permanent infertility is a real risk (66). Recently, a multicenter retrospective analysis of pregnancy and childbirth in patients who underwent AHSCT for different autoimmune diseases, including SSc, reported 15 pregnancies (68%) with healthy life births and no congenital, developmental or any other disease in the children. There were no reports with regard to maternal mortality associated with pregnancy or postpartum (67).

AHSCT-related late adverse events include malignancies (64). SSc patients who undergo myeloablative regimens receive TBI with lung and kidney shielding. TBI guarantees a stronger immune suppression in comparison to the non-myeloablative regimen. However, it is well known that in patients who have been given a TBI-based regimen there is a higher risk of secondary malignancies, particularly myelodysplasia/acute myeloid leukemia and later (only in trials with >10 years of follow-up) solid tumors (24, 25, 68). The relative long-term follow-up of transplanted SSc patients suggests that TBI is related to an increased risk of malignancies as shown by their onset in 9% of patients (two cases of myelodysplastic syndrome and one of medullary thyroid cancer) in the SCOT trial vs. 2 instances of EBV-positive lymphoproliferative disorder in the ASTIS trial (2.5%) (3, 4). It is interesting to note that, for unclear reasons, recent meta-analysis studies confirmed an increased incidence of cancer in SSc patients compared with the general population. The tumor types included lung cancer, non-Hodgkin's lymphoma and hematopoietic cancers (69, 70, 71). Thus, we can speculate that the intense immunosuppression related to transplantation might represent an additional risk factor for malignancies in SSc.

As observed in patients receiving HSCT for different indications (72), AHSCT, as do adverse events, can induce the onset of secondary autoimmune diseases. The AHSCT in autoimmune diseases is aimed at the immune system, inducing an intense immune-depletion and the consequent re-establishment of tolerance. However, during the immune reconstitution new autoreactive clones may arise and induce *de novo* autoimmunity (64, 73). In a retrospective EBMT registry analysis published in 2011, the incidence of secondary autoimmune disease was 9.8%. The most frequent secondary autoimmune diseases were organ-specific, including autoimmune thyroiditis, hemolytic anemia, autoimmune thrombocytopenia, and myasthenia gravis (64).

#### Treatment related mortality

Despite the proven efficacy of AHSCT in the treatment of SSc, transplant-related mortality (TRM) still represents a thorny issue and makes it difficult to view AHSCT as a standard therapy rather than a salvage option for the early and rapidly progressive forms of SSc.

AHSCT was performed safely in the first published RCT comparing transplantation with cyclophosphamide (ASSIST) (2). This trial enrolled a small number of patients (19), 10 of whom were allocated to receive AHSCT, with a follow-up of at least 2 years. The results were extremely positive in terms of efficacy and eight control patients, who progressed or did not improve by 1 year, were allowed to crossover at the AHSCT arm. Notably, no death or serious adverse events were registered during the study (2). Other studies failed to demonstrate such a high level of safety (74, 75). A retrospective analysis of a large cohort of SSc patients treated with the same non-myeloablative ASSIST regimen, reported a TRM of 6% (33) and this was mainly ascribable to cardiovascular complications. In the ASTIS trial, the TRM was 10% during the first year, and again cardiac events were suggested as the main cause of death (3). In the SCOT trial, TRM was lower than that previously reported (3% at 54 months and 6% at 72 months) and no deaths occurred during the first year (4). These differences in TRM in different AHSCT-RCTs may have multiple explanations and all of them essentially emphasize the key role of cardiac function in the safety of AHSCT in SSc. As a matter of fact, high dose cyclophosphamide is the agent most frequently associated with cardiac toxicity and preserved cardiac function is generally required for enrollment in clinical trials of high-dose chemotherapy (76). High-dose regimen of cyclophosphamide (4 gr/m^2^ in the mobilization phase and 200 mg/kg in the conditioning phase) used in the non-myeloablative AHSCT might be too toxic for those severe SSc patients, possibly with heart involvement. On the contrary, the SCOT trial, based on a low-dosage of cyclophosphamide, was not characterized by important cardiac events. Secondly, the low TRM reported in the studies by Burt et al. (2, 33) may be related to a more accurate cardiac evaluation. Similarly, none of the patients included in the SCOT trial had heart involvement or pulmonary hypertension (SCOT). Basal evaluation for the inclusion in the ASTIS trial consisted of echocardiogram for the detection of pulmonary hypertension and, only in this case, right heart catheterization was performed. However, it has been agreed that echocardiography cannot be reliable alone in making a diagnosis of pulmonary arterial hypertension. Right heart catheterization (RHC) is currently considered the gold standard for the evaluation of arterial pulmonary hypertension, further providing direct and accurate measurements of hemodynamics of the cardiovascular system (77). Furthermore, fluid challenge during RHC can give additional information to understand the cardiopulmonary response to increased volume load (as happens during AHSCT) and to identify patients with subclinical signs of SSc-related involvement of heart or pulmonary vasculature, neither of them being detectable at rest (33). Important complementary information may be provided by cardiac magnetic resonance imaging (CMR). CMR has emerged as the reference standard for assessment of left ventricular and right ventricular morphology, volumes and function (78) and it is considered a useful tool for the early assessment of cardiac involvement in SSc (79). The experience gained from a better knowledge of cardiac involvement in SSc and AHSCT studies is reflected in the current recommendations by the EBMT-Autoimmune Disease Working Party for a correct and extensive cardiopulmonary pre-transplant evaluation combining lung function tests, echocardiography, CMR imaging and invasive hemodynamic tests (80). Based on these recommendations, only patients without any evidence for PAH, even in the presence of fluid challenge during RHC, and good cardiopulmonary function can be considered for AHSCT.

Finally, both in the ASTIS and SCOT Trials, smoking status raised as an important element in compromising the AHSCT outcomes (3, 4). Compared to never smokers, previous and current smoking patients had a poorer overall survival. This finding is not new since it is common to other transplant setting (81), however the explanation of this correlation is largely speculative. Smoking is associated with impaired NK cytotoxic activity, unbalanced production of pro- and anti-inflammatory cytokines (81, 82) that may increase the risk of both respiratory and systemic infections in patients who experience compromised immunity. Furthermore, different studies provided strong evidence that tobacco use is detrimental to lung function in SSc patients further boosting abnormalities related to scleroderma lung disease (83–85).

In conclusion, in SSc patients, the risks of transplant-related complications and TRM are relatively high and depend on careful patient selection and evaluation, and the intensity of the transplantation regimen (agent and doses). These two features are strictly related to each other. Toxicity of the conditioning regimen largely depends on organ involvement and disease stage. In this perspective, the center experience and the close interspeciality networking provide an additional advantage (5).

### Recommendations

All the available studies prove the general concept of AHSCT as an effective, safe and feasible therapy in severe SSc. The potential of AHSCT to suppress, or at least ameliorate SSc features might be even more encouraging, if in the coming years the profile of the ideal SSc candidate for AHSCT will be better defined.

The criteria for the patient's selection and the timing of providing stem cell transplantation in patients with SSc are greatly needed. The key point is to identify patients with the highest possibility to have an improvement by the transplant procedure and the lowest risk of developing post-transplant life-threatening complications. The mortality risk of the disease being treated by AHSCT might justify the risk related to the transplant procedure. Several papers have concluded that many variables detected at the first evaluation of SSc patients are associated with reduced survival during long-term follow-up (55, 86, 87). These variables include male sex, older age, diffuse skin thickening, involvement of the heart, lung and kidney, and total skin thickness score. Domsic et al. (55) found that the rapid skin thickness progression rate (STPR) is an independent predictor of early mortality and the development of SRC. Observational studies have demonstrated that in diffuse scleroderma, most of the pathological processes in the internal organs or systems (gastrointestinal, lung, heart and kidney) occur within the first 3 years of the disease onset. Keeping these considerations in mind, patients with early and rapidly progressive diffuse SSc with evidence of at least mild involvement of heart, lung or kidney are the best candidates for AHSCT (Table 4). Thus, a more accurate cardiac assessment before directing a patient to AHSCT is certainly needed. Right catheterization with fluid challenge and cardiac magnetic resonance evaluation are the diagnostic tools proposed for this purpose (80). The lower rate of treatment-related mortality reported in most of the studies recently published, can probably be explained by the exclusion of patients with severe organ involvement. Certainly, one can consider that most trials have chosen arbitrary cut-offs for respiratory and cardiac function, and data are not so convincing for these measures. Anyway, it would be considered prudent to avoid patients with endstage lung disease to minimize infectious complications related to the different immunosuppressant drugs used during the mobilization and conditioning prior to AHSCT. Likewise, the presence of advanced heart disease may increase the risk of poor outcome in situations requesting higher heart performance as fever, infections and liquid overload infusion related to the transplant procedure.

**Table 4:** Main indications and contraindications to transplantation.

**Indications to AHSCT**	**Contraindications to AHSCT**
Acute onset	Long standing disease
Rapidly progressive disease refractory to conventional therapy	Indolent course of the disease
Mild organ damage	Irreversible organ involvement

A large body of evidence suggests the concept that, as for other rheumatic diseases, SSc has flogistic and active flares too, and therapeutic options, including AHSCT, should be tailored by considering the phase of the disease. Measuring disease activity in SSc has been particularly difficult in comparison with other autoimmune diseases in which it is possible to easily differentiate flares from quiescent phases. In 2001, the European Scleroderma Study Group (EScSG) developed a preliminary activity index that was subsequently validated and endorsed by the European Scleroderma Trials and Research group (EUSTAR) (62, 88). Recently, Del Papa et al. showed that AHSCT in patients with rapidly evolving dcSSc is effective in lowering both disease activity and severity of skin involvement (60). The same study showed that the patients selected in the control group (treated with conventional therapies) with comparable levels of disease activity had a 5-year probability of survival of around 40% in comparison to the higher percentage observed in the AHSCT group (80%) (60). This figure certainly demonstrated that the decrease in the disease activity is effective in prolonging survival and preserving organ damage related to persistent active disease.

A recent retrospective analysis of the EBMT autoimmune disease working party recognizes the great importance of center experience in the AHSCT outcome in autoimmune diseases, and in particular in SSc (5). Given the low prevalence of SSc patients with a severe form of the disease, the difficulty in identifying patients with a poor prognosis in the early phases, the experience needed for a correct evaluation of organ involvement, the complexity of AHSCT in these patients, a real benefit can certainly be achieved by creating close interaction between hematologists, rheumatologists, cardiologists and pulmonologists.

## Conclusions

Evidences from trials suggest that AHSCT is more effective than conventional immunosuppressive therapies at inducing a better long-term survival, ameliorating skin thickening and stabilizing internal organ function in severe SSc. The patients who can likely benefit from AHSCT are those with a rapid progressive and diffuse skin involvement, persistent high levels of disease activity, and mild initial organ damage. Center experience and specialist expertise are further important factors for improving outcomes of AHSCT strategies. Positive results from the published trials for AHSCT in SSc raise questions and new prospects of transplant activities. These challenges include (1) the definition of an optimal regimen intensity and in order to decrease TRM; (2) the availability of biomarkers or gene profiles able to select patients most likely to benefit from AHSCT; (3) a longer follow-up to identify late-onset adverse events; (4) the opportunity of a post-transplant immunosuppression to reduce the risks of disease relapse post-transplantation.

## Author contributions

ND designed the plan of the review, revised the literature and wrote the paper. FP revised the recent literature and the final paper. EZ, WM, and AM revised the literature and the final version of the paper.

### Conflict of interest statement

The authors declare that the research was conducted in the absence of any commercial or financial relationships that could be construed as a potential conflict of interest.
